# Intravenous infusion of human umbilical cord Wharton’s jelly-derived mesenchymal stem cells as a potential treatment for patients with COVID-19 pneumonia

**DOI:** 10.1186/s13287-020-01725-4

**Published:** 2020-05-27

**Authors:** Yingxin Zhang, Jie Ding, Shaoda Ren, Weihua Wang, Yapei Yang, Shuangjing Li, Min Meng, Tiejun Wu, Daliang Liu, Suochen Tian, Hui Tian, Shuangfeng Chen, Changhui Zhou

**Affiliations:** 1 Department of Central Laboratory, Liaocheng People’s Hospital, Dongchang West Road, No.67, Liaocheng, 252000 Shandong Province China; 2 Department of ICU, Liaocheng People’s Hospital, Liaocheng, 252000 Shandong Province China; 3 Department of CT room, Liaocheng People’s Hospital, Liaocheng, 252000 Shandong Province China

**Keywords:** COVID-19, Human umbilical cord Wharton’s jelly-derived MSCs, Immunomodulatory, Treatment

## Abstract

The novel coronavirus disease 2019 (COVID-19) has grown to be a global public health emergency since patients were first detected in Wuhan, China. Thus far, no specific drugs or vaccines are available to cure the patients with COVID-19 infection. The immune system and inflammation are proposed to play a central role in COVID-19 pathogenesis. Mesenchymal stem cells (MSCs) have been shown to possess a comprehensive powerful immunomodulatory function. Intravenous infusion of MSCs has shown promising results in COVID-19 treatment. Here, we report a case of a severe COVID-19 patient treated with human umbilical cord Wharton’s jelly-derived MSCs (hWJCs) from a healthy donor in Liaocheng People’s Hospital, China, from February 24, 2020. The pulmonary function and symptoms of the patient with COVID-19 pneumonia was significantly improved in 2 days after hWJC transplantation, and recovered and discharged in 7 days after treatment. After treatment, the percentage and counts of lymphocyte subsets (CD3^+^, CD4^+^, and CD8^+^ T cell) were increased, and the level of IL-6, TNF-α, and C-reactive protein is significantly decreased after hWJC treatment. Thus, the intravenous transplantation of hWJCs was safe and effective for the treatment of patients with COVID-19 pneumonia, especially for the patients in a critically severe condition. This report highlights the potential of hWJC infusions as an effective treatment for COVID-19 pneumonia.

## Introduction

The novel coronavirus disease 2019 (COVID-19) has grown to be a global public health emergency since patients were first detected in Wuhan, China, in December 2019 [1, 2]. The 2019 novel coronavirus had infected 418,209 people worldwide (among which 18,724 were killed) as of 25 March, 2020. Including the ground-glass opacity in the lung, the other typical diagnosis characteristic of critically ill patients was a significant decrease in lymphocytes along with the increase of neutrophils [2–5]. Currently, no specific drugs or vaccines are available to cure patients with COVID-19 infection [2, 6]. Therefore, novel strategies for critically ill COVID-19 are urgently needed. Critically ill patients have higher concentrations of interleukin-6 (IL-6), granulocyte colony-stimulating factor (G-CSF), and tumor necrosis factor-α (TNF-α), indicating the virus can stimulate a terrible cytokine storm in the lung, which may cause severe organ injury and death [5–8]. Therefore, avoiding the cytokine storm may be the key for the treatment of COVID-19-infected patients [6, 9].

Mesenchymal stem cells (MSCs) have been widely used to treat autoimmune disease, graft-versus-host disease (GVHD), and other diseases with very good safety [10, 11]. MSCs play a positive role in immunomodulatory effects via secreting many types of cytokines by paracrine secretion or make direct interactions with immune cells [3, 11]. Among which, the human umbilical cord Wharton’s jelly-derived MSCs (hWJCs) can be easily obtained and cultured. hWJCs have shown very significant immunomodulation and tissue repair effects with low immunogenicity, which makes them a very ideal candidate for allogeneic adoptive transfer therapy [3, 11]. Owing to their powerful immunomodulatory ability, hWJC transplantation may have beneficial effects on preventing or attenuating the cytokine storm [6, 9, 12, 13].

Up to now, the therapeutic effects of hWJCs on COVID-19 is still rarely reported. Here, we will introduce a critically ill elder male patient in China infected with COVID-19. The clinical outcome of hWJC adoptive transfer therapy will be discussed to explore their therapeutic potential for COVID-19-infected patients.

## Patient and methods

The study was conducted in Liaocheng People’s Hospital, China, and approved by the ethics committee of the hospital (2020003, Supplementary Figure 1). This patient without malignant tumors and have not participated in other clinical trials within 3 months.

## Case presentation

On February 7, 2020, a 54-year-old man presented to Yanggu People’s Hospital, Shandong, with a 4-day history of cough, chest tightness, and fever. Apart from a 2-year history of diabetes, the patient had no other specific medical history. The physical examination showed a body temperature of 38.0 °C, blood pressure of 141/87 mmHg, and pulse of 81 beats per minute. A blood routine examination was arranged urgently, and throat swabs were collected. The result revealed that the white cell count and absolute lymphocyte count were 7.59 × 10^9^/L (reference range 3.5~9.5 × 10^9^/L) and 0.24 × 10^9^/L (reference range 1.1~3.2 × 10^9^/L), respectively; C-reactive protein (CRP), 59.64 mg/L (reference range 0 ~ 10 mg/L); influenza A and B virus antigen (−); and routine anti-inflammation and antivirus therapy were given for supportive treatment.

On February 9, 2020, the real-time polymerase chain reaction (RT-PCR) assay confirmed that the patient’s specimen tested positive for COVID-19. Then, the patient was admitted to an airborne isolation unit in Liaocheng Infectious Disease Hospital for clinical observation.

On February 11, 2020, the patient felt severe shortness of breath, and the oxygen saturation values decreased to as low as 87.9%. Related laboratory results showed PH (7.46), PCO_2_ (26 mmHg), PO_2_ (50 mmHg), HCO_3_ (18.4 mmol/L). The doctors decided to change the diagnosis to COVID-19 (critically severe type), and the patient was admitted to the ICU of Liaocheng People’s Hospital for better treatment.

**Fig. 1:**
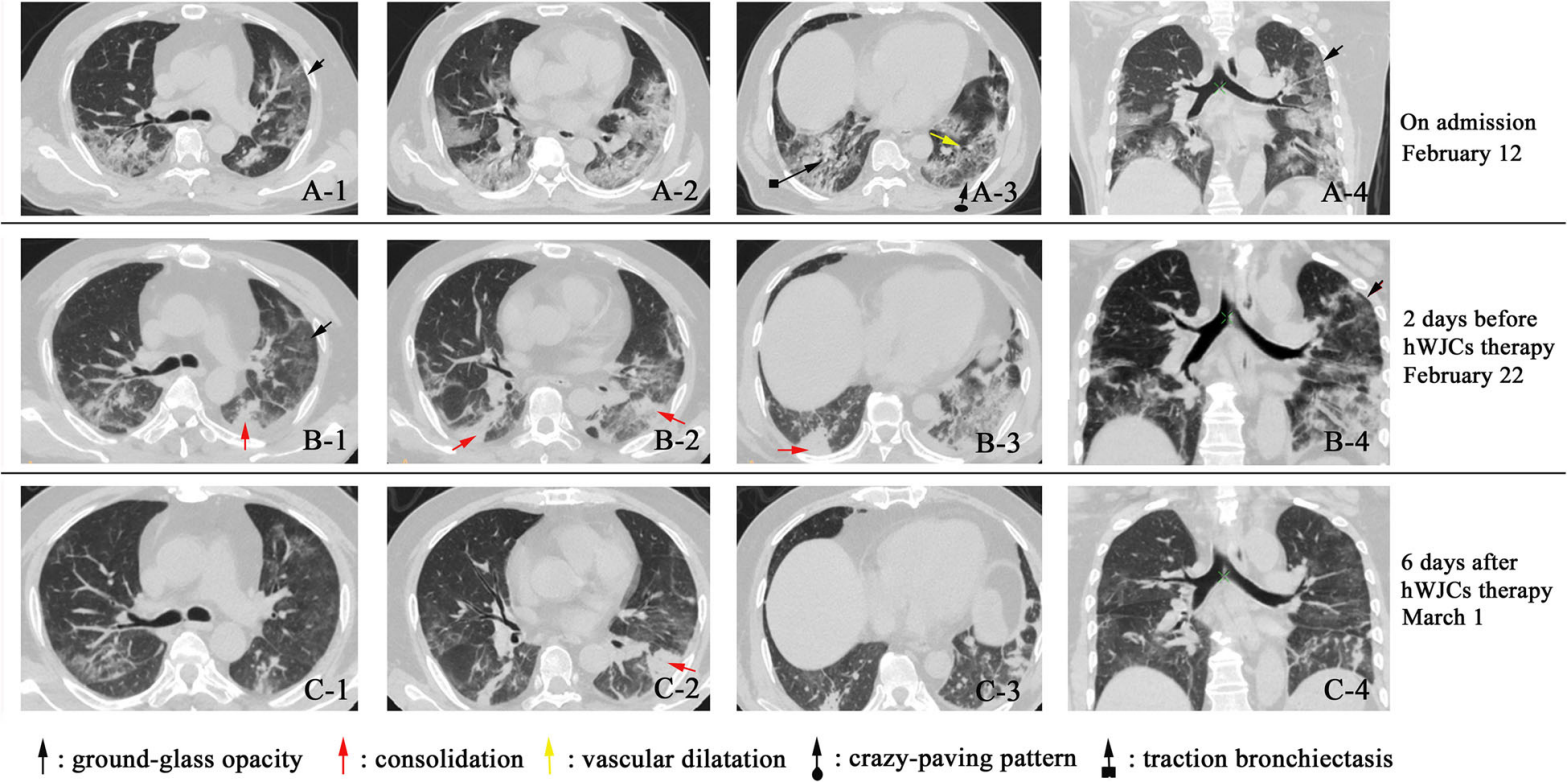
Chest computerized tomography (CT) images of the COVID-19 patient. **A-1**–**A-4** On February 12, ground-glass opacity (GGO) and pneumonia infiltration occurred in both the left and right lungs. Several GGO regions in each of the 5 lung lobes, and some with traction bronchiectasis; in the left lower lobe, crazy-paving pattern (GGO with superimposed inter- and intralobular septal thickening) with a few scattered consolidation and vascular dilatation were observed. **B-1**–**B-4** CT images on February 22 indicate the symptoms of the patient are slightly relieved, but the pneumonia was still significant. There were reduced regions of initial GGO, with a new area of subpleural consolidation. **C-1**–**C-4** Cell transplantation was performed on February 24. On March 1, the pneumonia infiltration faded away very much. Most of the ground-glass opacity lightened, or even disappeared. The partial area of consolidation was still observed

On February 12, 2020, the shortness of breath even got worse under the oxygen supplementation. The doctor speeded up the oxygen airflow to 45 L per minute. Chest computerized tomography (CT) clearly showed evidence of pneumonia and ground-glass opacity, in the right and left lungs (Fig. 1A-1–A-4). According to the guideline for the diagnosis and treatment of COVID-19 [14], the patient was treated with antiviral therapy of lopinavir/ritonavir, IFN-α inhalation, and also intravenous injection of levofloxacin, tanreqing capsule, xuebijing, thymosin α1, methylprednisolone, and immunoglobulin. During this time, the patient received antipyretic therapy. More treatments were conducted consisting of electrocardiograph monitoring, potassium chloride sustained-release tablets (oral, 1 g per time, 2 times per day), plasma exchange and regulated intestinal microflora of patient, etc. Finally, the discomfort was released, and the oxygen saturation increased to 98%.

On February 13 to 21, the patient’s vital physical signs remained largely stable, apart from the development of intermittent fevers and shortness of breath.

On February 22, the patient took a turn for the worse (Fig. 1B-1–B-4). Considering the severe organ injury caused by an inflammatory response, hWJC adoptive transfer therapy was proposed under the advice and guidance of the specialist group. The family member and patient agreed to try hWJC adoptive transfer therapy. The therapeutic scheme was then discussed and approved by the ethics committee of the hospital, and consent forms were signed by the family member before the therapy. On February 24, the patient receives hWJC transfusion.

On March 1, the patient felt much better. The shortness of breath was significantly recovering. The CRP decreased to 27.2 g/L, the absolute lymphocyte count rose to 0.66 × 10^9^/L, and the inflammatory factors reduced to normal levels, which indicated that the patient was recovering rapidly. On March 2, the patient meets the discharge standard, and the medical observation is canceled

## hWJC preparation

The clinical-grade hWJCs were supplied by the Central Laboratory of Liaocheng People’s Hospital. The cell product has been certified by the National Institutes for Food and Drug Control of China (authorization number: SH201900594 and SH201900597).

hWJCs were expanded as previously described [11]. Briefly, following the standard operating procedures, human umbilical cords were obtained under sterile conditions from full-term infants delivered by cesarean section; residual blood was fully washed by phosphate-buffered saline. The umbilical cord membrane was stripped, and the umbilical cord blood vessels (two arteries and one vein) were removed to retain the Wharton’s jelly. The Wharton’s jelly was cut into 1-mm^3^ pieces and then cultured in DMEM/F12 (GIBCO, USA) supplemented with 10% fetal bovine serum (FBS) (GIBCO, USA). The culture was maintained at 37 °C with saturated humidity and 5% (v/v) CO_2_. The medium was changed every 3 days. hWJCs were passaged at 80% confluence treatment with 0.25% trypsin (Sigma, USA) and 0.02% EDTA (Sigma, USA).

## hWJC characterization and transplantation

hWJCs were characterized by flow cytometric analysis of hWJC-associated cell surface markers, as previously described [11]. hWJC-associated cell surface marker analysis was performed by incubating 200,000 hWJCs with a combination of antibodies: CD90-fluorescein isothiocyanate (FITC, 555595), CD73-phycoerythrin (PE, 550257), CD105-phycoerythrin (PE, 560839), CD45-(APC, 555485), and CD34-phycoerythrin-Cy (PE-Cy, 5555823). All were manufactured by Becton-Dickinson BD, San Diego, CA, and hWJCs were analyzed using a FACS Canto cytometer (BD Biosciences, San Jose, CA) and the Diva Software (BD Biosciences, San Jose, CA).

Release testing before administration comprised morphology (fibroblast-like adherent cells), viability (> 90% live cells measured by trypan blue assay), purity by FACS (≥ 95% CD90^+^, CD73^+^, CD105^+^, CD34^−^, and CD45^−^ cells), endotoxin and mycoplasma and sterility testing (−), and cell viability test (> 90%).

The hWJCs injected to the patient were from fresh culture. Patients received dexamethasone 2 mg before intravenous infusion of hWJCs. hWJCs were suspended in 100 mL of normal saline, and the total number of transplanted cells was calculated by 1 × 10^6^ cells per kilogram of weight. The injection was performed about 40 min with a speed of 40 drops per minute. During hWJC injection, other conventional therapies are used as usual.

The patient was assessed by the investigators after receiving the investigational product. The clinical, laboratory, and radiological outcomes were recorded and certified by a trained group of doctors. The detailed record included primary safety data (infusional and allergic reactions, secondary infection, and life-threatening adverse events) and the efficacy outcomes mainly included the level of the cytokine variation, the level of CRP in the plasma, the lymphocyte subpopulations, the chest CT, and the patient symptoms.

## COVID-19 nucleic acid detection

RT-PCR analysis of COVID-19 nucleic acid was performed before and after hWJC transplantation, as previously described [15]. Briefly, viral RNA was extracted from 200 μL of respiratory specimens from patients suspected of having COVID-19 infection using the Nucleic Acid Extraction Kit (TianLong, Suzhou, China) according to the manufacturer’s recommended procedure. RT-PCR assay was performed using a COVID-19 nucleic acid detection kit according to the manufacturer’s protocol (Shanghai BioGerm Medical Technology Co Ltd., national medical device registration approval number: 20203400065). It was performed under the following conditions: incubation at 50 °C for 10 min and 95 °C for 5 min, 40 cycles of denaturation at 95 °C for 10 s, and extending and collecting fluorescence signal at 55 °C for 40 s. A cycle threshold value (Ct value) less than 38 was defined as a positive test result, and a Ct value of 40 or more was defined as a negative test.

## Results

### Safety outcome

Before the hWJC transplantation, the patients had symptoms of high fever (38.5 °C ± 0.5 °C), weakness, shortness of breath, and low oxygen saturation. However, 2~7 days after transplantation, all the symptoms disappeared, and the oxygen saturations rose to 98% at rest. In addition, no acute infusion-related or allergic reactions were observed within 2 h after transplantation. Similarly, no delayed hypersensitivity or secondary infections were detected after treatment.

### The efficacy outcome

Both fever and shortness of breath disappeared on the 2nd day after hWJC transplantation. Chest CT imaging showed that the ground-glass opacity and pneumonia infiltration had largely reduced on the 6th day after hWJC transplantation (Fig. 1C-1–C-4). The CT score of the pulmonary involvement [16] was represented in Table 1.

**Table 1:** The CT score of the pulmonary involvement

Date	Position of the lung, score				
February 12	Left upper lobe, 2	Left lower lobe, 3	Right upper lobe, 1	Right middle lobe, 1	Right lower lobe, 3
February 22	Left upper lobe, 3	Left lower lobe, 4	Right upper lobe, 1	Right middle lobe, 1	Right lower lobe, 3
March 1	Left upper lobe, 1	Left lower lobe, 2	Right upper lobe, 1	Right middle lobe, 1	Right lower lobe, 2

A semi-quantitative scoring system was used to quantitatively estimate the pulmonary involvement of all abnormalities on the basis of the area involved [16]. Each of the 5 lung lobes was visually scored from 0 to 5 as follows: 0, no involvement; 1, < 5% involvement; 2, 25% involvement; 3, 26–49% involvement; 4, 50–75% involvement; and 5, > 75% involvement

**Fig. 2:**
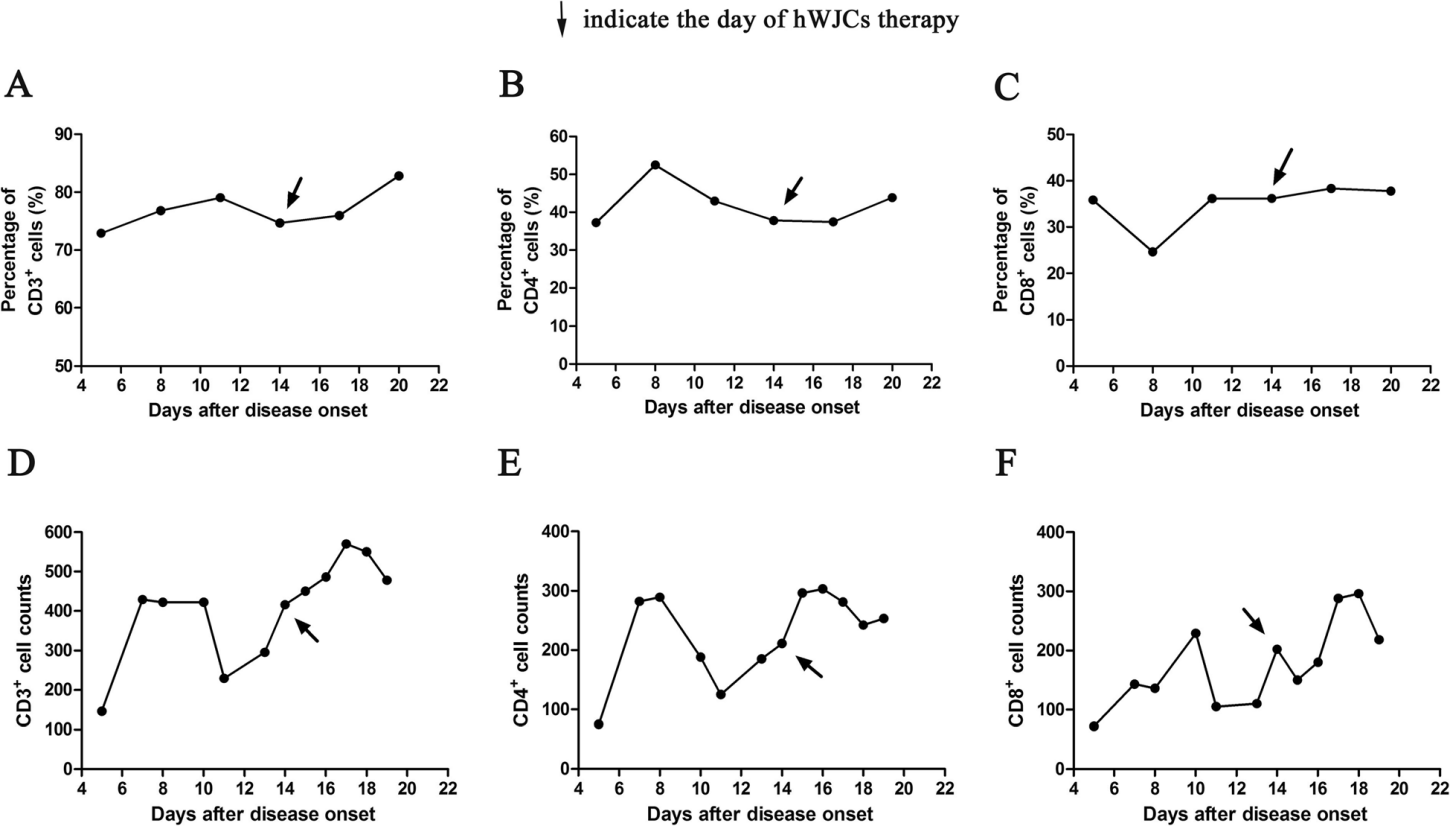
The dynamic changes of the lymphocyte subsets of the patient. The percentage of CD3^+^ T cell (**a**), CD4^+^ T cell (**b**), and CD8^+^ T cell (**c**) and the counts of CD3^+^ T cell (**d**), CD4^+^ T cell (**e**), and CD8^+^ T cell (**f**) were all increased after intravenous injection of hWJCs

**Fig. 3:**
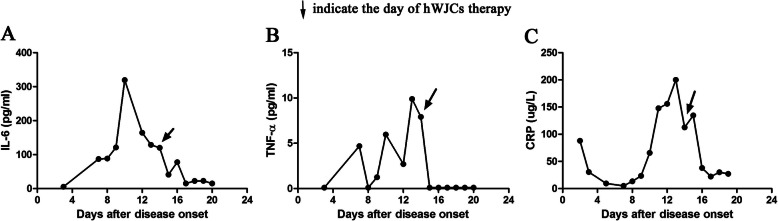
The dynamic changes of IL-6 (**a**), TNF-α (**b**), and C-reaction protein (**c**). The level of plasma C-reaction protein, IL-6, and TNF-α was significantly decreased after intravenous injection of hWJCs

The immunoregulatory function of hWJCs contributed to the main efficacy outcome. As shown in Fig. 2, the percentage and counts of CD3^+^ T cell, CD4^+^ T cell, and CD8^+^ T cell were increased, which indicated that the immune function of patients was improved after the hWJC administration. Meanwhile, the levels of plasma CRP and inflammatory factors (IL-6 and TNF-α) were all decreased after hWJC treatment (Fig. 3). All these results indicated that the inflammation status of the patient was alleviating quickly.

For the critically severe patient, before hWJC transplantation, COVID-19 nucleic acid was positive. Six days after transplantation (March 1), COVID-19 nucleic acid turned to be negative.

### Discussion

The cure of COVID-19 is essentially dependent on the patient’s own immune system. The virus infection caused a total function failure of the lymphocytes, even of the whole immune system [6, 17]. However, when the overactivated immune system kills the virus, it produces a large amount of inflammatory factors, leading to severe cytokine storms [6, 15]. The cytokine storms deteriorated the patient’s states which may cause the disabled function of endothelial cells and the capillary leakage and could lead to multiple organ failure [3, 6].

MSC therapy can inhibit the overactivation of the immune system and promote endogenous repair by improving the microenvironment [6]. After entering the human body through intravenous infusion, part of the MSCs accumulates in the lung, which could improve the pulmonary microenvironment, protect alveolar epithelial cells, prevent pulmonary fibrosis, and improve lung function [12, 13]. Meanwhile, recent studies suggest that MSCs played the vital immune modulation roles on lymphocyte subsets and can secrete anti-inflammatory factors to prevent the cytokine storm [6, 9, 11].

Our results showed that the administration of intravenous injection of hWJCs significantly improved the inflammation and immune situation in severe COVID-19 patients. After hWJC adoptive transfer, no obvious side effects were observed, indicating it was well tolerated. The serum CRP and inflammatory factors (IL-6 and TNF-α) of the patient were gradually reduced, and some other vital signs were also improved. The trachea cannula was also pulled off, and the patient could ambulate on the ground from February 26 as well. Meanwhile, the counts of CD3^+^, CD4^+^, and CD8^+^ T cell were remarkably increased after intravenous injection of hWJCs. All these results suggested that transplantation of hWJCs could improve the outcome of COVID-19 patients maybe through regulating inflammatory response and promoting the recovery of antiviral immune cells and organs.

In addition, emerging evidences indicate that the epithelial and endothelial regulated cell death (RCD) caused by inflammation is closely related to the pathogenesis of many pulmonary disorders [18]. MSCs are known to improve cell survival and prevent apoptosis, necroptosis, and pyroptosis occurring in various parenchymal or nonparenchymal cells and immune cells under unfavorable conditions [18, 19]. The assessment of the ability of hWJCs in modulating RCD would certainly benefit patients receiving hWJC therapy, particularly those with COVID-19. Therefore, how hWJCs can counteract cell death and promote cell regeneration should be discussed in the future.

## Conclusions

As a conclusion, we proposed that the adoptive transfer therapy of hWJCs might be an ideal choice to be used for COVID-19 treatment. Although only one case was shown here, it would also be very important to inspire more similar clinical practice to treat such critically ill COVID-19 patients.

## Supplementary information

**Additional file 1: Supplementary Figure 1.** Ethical approval of hWJCs for the treatment of patients with COVID-19 pneumonia.

## Data Availability

The datasets used and/or analyzed during the current study are available from the corresponding authors on reasonable request.

## Abbreviations

COVID-19: Novel coronavirus disease 2019
IL-6: Interleukin-6
G-CSF: Granulocyte colony-stimulating factor
TNF-α: Tumor necrosis factor-α
MSCs: Mesenchymal stem cells
hWJCs: Human umbilical cord Wharton’s jelly-derived mesenchymal stem cells
GVHD: Graft-versus-host disease
CRP: C-reactive protein
RT-PCR: Real-time polymerase chain reaction
CT: Computerized tomography
GGO: Ground-glass opacity
FBS: Fetal bovine serum
RCD: Regulated cell death
