# Supplementary figures and images for: Intravenous infusion of human umbilical cord Wharton’s jelly-derived mesenchymal stem cells as a potential treatment for patients with COVID-19 pneumonia
Source: Stem Cell Res Ther. 2020 May 27;11:207. doi: 10.1186/s13287-020-01725-4 (PMC7251558; doi:10.1186/s13287-020-01725-4)

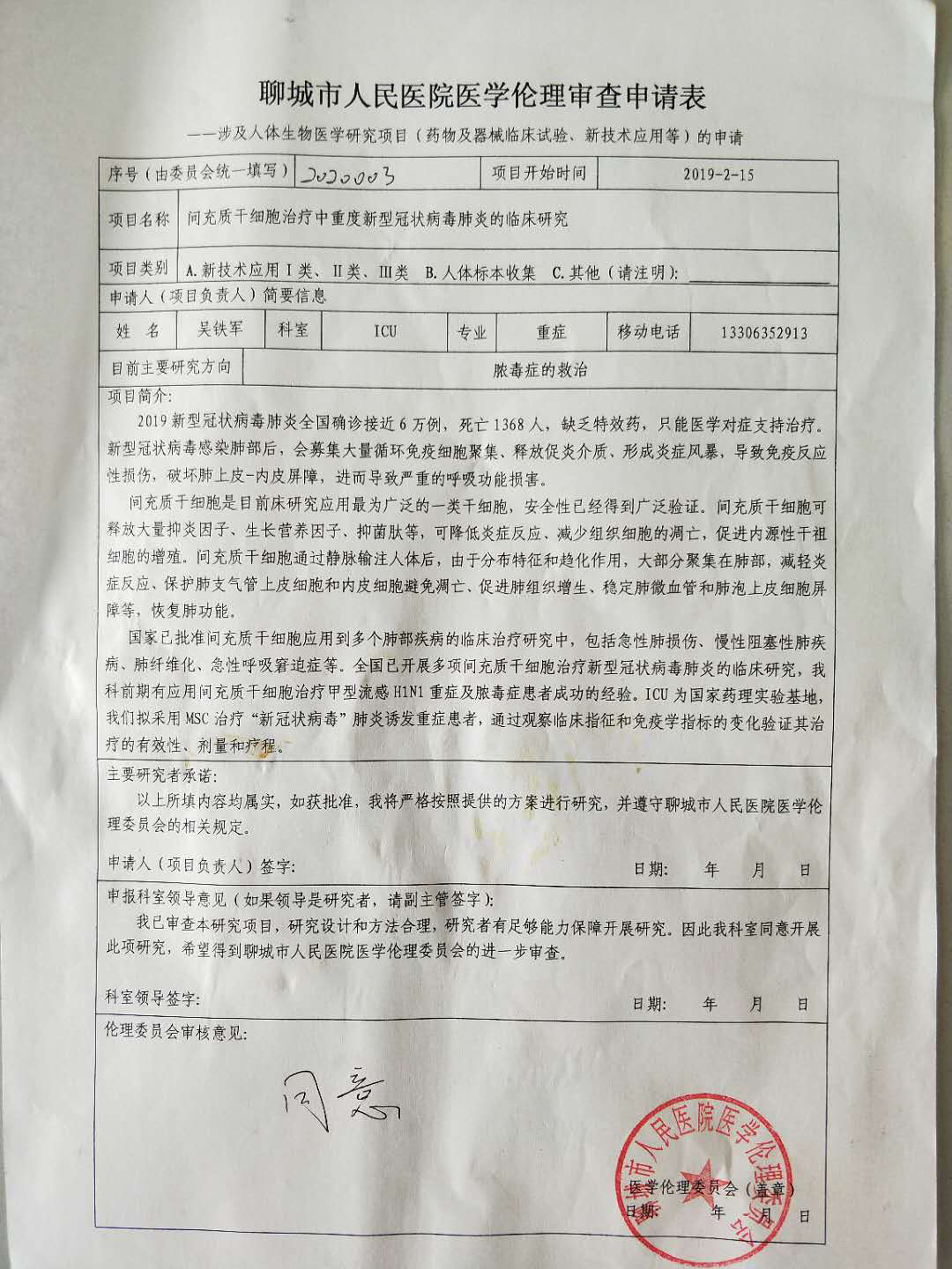

Supplement: Supplementary file 1 — Additional file 1: Supplementary Figure 1. Ethical approval of hWJCs for the treatment of patients with COVID-19 pneumonia. [file 13287_2020_1725_MOESM1_ESM.jpg]
